# Supplementary material for: Mapping the evolution of fertility support policies in China: A content and instrumental analysis
Source: PLoS One. 2025 Oct 9;20(10):e0332137. doi: 10.1371/journal.pone.0332137 (PMC12510515; doi:10.1371/journal.pone.0332137)
Supplement: S1 Appendix — (ZIP) [file pone.0332137.s001.zip › S1 Appendix. 226 original policy documents/89-教育部办公厅等七部门关于教育支持社会服务产业发展 提高紧缺人才培养培训质量的意见(FBM-CLI-4-336414).doc]

教育部办公厅等七部门关于教育支持社会服务产业发展 提高紧缺人才培养培训质量的意见

**发布部门：** [教育部](https://www.pkulaw.com/law/chl?Aggs.IssueDepartment=60203) [国家发展和改革委员会(含原国家发展计划委员会、原国家计划委员会)](https://www.pkulaw.com/law/chl?Aggs.IssueDepartment=60301) [民政部](https://www.pkulaw.com/law/chl?Aggs.IssueDepartment=60208) [商务部](https://www.pkulaw.com/law/chl?Aggs.IssueDepartment=60220) [国家卫生健康委员会](https://www.pkulaw.com/law/chl?Aggs.IssueDepartment=60321) [国家中医药管理局](https://www.pkulaw.com/law/chl?Aggs.IssueDepartment=7060306) [全国妇女联合会](https://www.pkulaw.com/law/chl?Aggs.IssueDepartment=70403) [机构沿革](javascript:void(0))

**发文字号：**教职成厅〔2019〕3号

**发布日期：**2019.09.05

**实施日期：**2019.09.05

**时效性：** [现行有效](https://www.pkulaw.com/law/chl?Aggs.TimelinessDic=01)

**效力级别：** [部门规范性文件](https://www.pkulaw.com/law/chl?Aggs.EffectivenessDic=XE0302)

**法规类别：** [职业与职工教育](https://www.pkulaw.com/law/chl?Aggs.Category=09123)

教育部办公厅等七部门关于教育支持社会服务产业发展 提高紧缺人才培养培训质量的意见
（教职成厅〔2019〕3号）

各省、自治区、直辖市教育厅（教委）、发展改革委、民政厅（局）、商务厅（局、委）、卫生健康委、中医药管理局、妇联，新疆生产建设兵团教育局、发展改革委、民政局、商务局、卫生健康委、妇联：
　　社会服务产业是涉及亿万群众福祉的民生事业和具有巨大发展潜力的朝阳产业，大力发展社会服务产业对更好满足人民群众日益增长的美好生活需要、高水平全面建成小康社会具有重要意义。为贯彻党中央、国务院关于促进家政服务业提质扩容、推进养老服务发展、促进婴幼儿照护服务发展等的决策部署，落实《国家职业教育改革实施方案》等，加快推进社会服务产业人力资源供给侧结构性改革，教育部、国家发展改革委、民政部、商务部、国家卫生健康委、国家中医药局、全国妇联办公厅就教育支持社会服务产业发展，提高家政、养老、育幼等领域紧缺人才培养培训质量提出如下意见。
　　**一、**总体要求
　　（一）指导思想
　　以习近平新时代中国特色社会主义思想为指导，全面贯彻党的十九大精神，落实全国教育大会精神，坚持以人民为中心，贯彻党的教育方针，主动适应家政服务业与养老、育幼、物业、快递等融合发展新模式，居家为基础、社区为依托、机构为补充、医养相结合的养老服务体系建设新要求，家政电商、“互联网＋家政”“物业＋养老服务”“互联网＋养老”等新业态，不断满足城乡社区居民多样化、个性化、中高端新需求，以社区为重点依托，聚焦专业人才供给，拓展社会服务产业发展空间，以职业教育为重点抓手，提高教育对社会服务产业提质扩容的支撑能力，加快建立健全家政、养老、育幼等紧缺领域人才培养培训体系，扩大人才培养规模，全面提高人才培养质量，支撑服务产业发展，增强广大人民群众的获得感、幸福感和安全感。
　　（二）基本原则
　　政府主导，协调发展。加强统筹规划，将社会服务产业紧缺领域人才培养培训工作与学科专业调整，招生、培养、就业联动机制建设，教育脱贫攻坚等同步设计，优先部署，促进协调发展。
　　对接需求，分类施策。针对行业发展不同领域、不同模式、不同业态对人才的差异化需求，以服务家政服务、健康管理、养老照护、母婴照护等一线高素质技术技能人才为重点，兼顾考虑储备社会服务新业态急需人才，分层分类推进培养培训。
　　育训结合，统筹推进。坚持学历教育与职业培训并举并重，统筹推进专业设置、课程体系建设、师资队伍建设、学生资助、实习实训基地建设等人才培养培训各环节，提高专业人才供给规模和质量。
　　（三）工作目标
　　到2022年，教育支持社会服务产业发展的能力有效增强，紧缺领域相关学科专业体系进一步完善，结构进一步优化，布局进一步拓展，培养培训规模显著扩大，内涵进一步提升，教师教材教法改革、产教融合校企合作不断深化，为社会服务产业紧缺领域培养和输送一大批层次结构合理、类型齐全、具有较高职业素养和专业能力的高素质人才。

　　**二、**任务措施
　　1.完善学科专业布局。健全专业随产业发展的动态调整机制，调整优化学科专业目录，及时增设相关领域本专科专业。以面向社区居民的家政服务、养老服务、中医药健康服务、托育托幼等紧缺领域为重点，对接管理、经营、服务、供应链等岗位需求，合理确定中职、高职、本科、研究生等不同类型、层次学历教育相关专业和职业培训的人才培养目标、规格。在一流本科专业建设“双万计划”、中国特色高水平高职学校和专业建设计划等项目实施过程中，向家政、养老、育幼等相关领域专业倾斜。
　　2.重点扩大技术技能人才培养规模。鼓励引导有条件的职业院校积极增设护理（老年护理方向、中医护理方向）、家政服务与管理、老年服务与管理、智能养老服务、健康管理、中医养生保健、中医营养与食疗、助产、幼儿发展与健康管理、幼儿保育、学前教育、康复治疗技术、中医康复技术、康复辅助器具技术、康养休闲旅游服务、健身指导与管理等社会服务产业相关专业点。鼓励院校根据医养结合、安宁疗护、心理慰藉、家庭理财、收纳管理、服饰搭配和衣物管理、室内适老化设计、社区服务网点规划设计等产业发展新岗位、新需求，灵活设置专业方向。每个省份要有若干所职业院校开设家政服务、养老服务类专业，引导围绕社会服务产业链打造特色专业群。扩大中高职贯通培养招生专业和规模。引导应用型本科高校、本科层次职业教育试点院校开设相关专业，加快培养高端家政服务人才，养老机构、家政机构、大型康养综合体经营管理等急需人才。
　　3.加快培养适应新业态、新模式需要的复合型创新人才。鼓励引导普通本科高校主动适应社会服务产业发展需要，设置家政学、中医康复学、中医养生学、老年医学、康复治疗学、心理学、护理学和社会工作等相关专业。原则上每个省份至少有1所本科高校开设家政服务、养老服务、托育服务相关专业。鼓励普通本科高校电子信息类、机械类、材料类等专业，高职院校电子信息大类、装备制造大类等专业增设相关课程，加快培养家庭服务机器人、健康监测、家用智能监控等健康养老、家政服务领域智能设施设备的研发制造人才，促进人工智能技术、虚拟现实（VR）技术、智能硬件、新材料等在社会服务业深度应用。在普通本科高校金融学类、高职院校财经商贸大类专业中增设相关课程，不断满足养老金融创新急需人才。鼓励有条件的普通高校探索辅修专业或双学士学位等培养模式，加快培养社会服务产业相关领域管理和培训人才。
　　4.积极培养高层次管理和研发人才。加强社会服务业相关学科基础科研。支持高校通过自设家政学等二级学科，开展相关产业政策研究和人才培养。促进相关交叉学科专业发展，服务以专业设备、专用工具、智能产品研发制造为支持的家政服务产业集群建设。鼓励和支持有条件的高校在相关学科领域招收培养研究生，为企业和职业院校等输送业务骨干和高层次教学科研人员。
　　5.支持从业人员学历提升。鼓励符合条件的家政服务、养老服务企业、养老服务机构管理人员报考攻读专业学位硕士研究生。支持社会服务产业从业人员通过多种渠道接受职业教育，提升学历。开放大学要充分发挥办学优势，加快信息化学习资源和平台建设，探索建立面向社会服务产业从业人员的现代远程教育教学及支持服务模式。
　　6.鼓励院校广泛开展职业培训。推动职业院校联合相关企业，促进企业职工岗位技术技能水平提升。支持职业院校发挥资源优势，重点为困难企业转岗职工、去产能分流职工和贫困劳动力等就业重点人群从事社会服务产业提供职业培训，承担“雨露计划”“巾帼家政服务培训”“家政培训提升行动”等培训任务。鼓励职业院校联合行业企业共同开展市场化社会培训。
　　7.健全教学标准体系。发挥标准在人才培养培训质量提升中的基础性作用。按照专业设置与产业需求对接、课程内容与职业标准对接、教学过程与生产过程对接的要求，持续更新并推进社会服务产业领域职业院校专业教学标准、顶岗实习标准、实训教学条件建设标准等的建设和实施。推进有关本科专业类教学质量标准实施。指导院校贯彻落实国家教学标准，按照有关要求科学制订和实施人才培养方案，保障人才培养质量。
　　8.建设高质量课程教材资源。注重强化职业道德、职业素养、安全意识、法治教育，有关专业课程重点向老年服务与管理、病患护理、母婴照料等领域倾斜，适度拓展心理学、医学、营养学、沟通技巧等基础知识。在国家规划教材建设中，加大社会服务产业紧缺领域相关专业教材建设支持力度，遴选200种校企双元开发的优质教材，倡导新型活页式、工作手册式教材。鼓励有关院校引入企业真实项目和案例，开发或引入多种形式的数字化教学资源，在职业教育专业教学资源库建设中向相关专业倾斜，做好老年服务与管理、学前教育专业教学资源库的更新和使用工作。
　　9.开展1＋X证书制度试点。积极招募、推动职业教育培训评价组织联合社会服务产业优质企业、职业院校共同研制家政服务、养老服务、母婴照护等紧缺领域职业技能等级标准和证书，开发教材和学习资源。支持院校学生在获得学历证书的同时，积极取得紧缺领域相关职业技能等级证书，提高就业创业本领，促进高质量就业。在家政服务、养老服务、托育服务等领域率先开展1＋X证书制度试点，同步探索建设职业教育国家学分银行。
　　10.推动校企深度合作。鼓励社会力量举办家政服务类、养老服务类职业院校，或与职业院校以股份制、混合所有制等形式共建产业学院，合作开设相关专业，规范并加快培养专门人才。将社会服务产业紧缺领域列为校企合作重点领域，优先支持建设产教融合创新项目、职业教育校企深度合作项目等。全国建设培育100家以上产教融合型家政企业，发挥家政服务业提质扩容“领跑者”行动示范企业和普惠养老重点企业的示范引领作用，推动50家优质企业与200所有关院校组建职业教育集团等，共建产业学院、大师工作室、协同创新平台、实习实训基地，实行现代学徒制、“订单培养”等培养模式，协同创新服务项目或开展技术研发，支持和鼓励企业承接教师实践锻炼和学生见习实习，深度参与紧缺领域人才培养培训。
　　11. 鼓励学生创新创业。鼓励院校围绕“互联网＋家政”“互联网＋养老”“互联网＋健康服务”等，建设众创空间，指导学生开展自主创新和创业活动，做好创业项目的跟踪、指导和孵化服务，引导有条件的学生积极投入社会服务产业相关领域创业。支持鼓励相关专业学生参加中国“互联网＋”大学生创新创业大赛。在全国职业院校技能大赛中论证设置相关特色赛项。相关院校要根据毕业生特点，加强职业指导和就业创业服务。组织相关专业学生到养老服务等公益属性较强的社会服务机构和城乡社区、家庭等开展社会实践活动。
　　12.打造“双师型”教师队伍。在职业院校实行高层次、高技能人才以直接考察的方式公开招聘，建立健全职业院校自主聘任兼职教师的办法。新增相关专业课教师原则上应从具备家政、养老服务、社区服务等工作经历人员中引入和选聘。优先支持社会服务相关专业领域符合项目式、模块化教学需要的职业教育教师教学创新团队。在职业院校教师素质提高计划中对相关专业予以重点推进。在“双师型”教师培养培训基地建设中向社会服务相关专业倾斜。依托职业院校、应用型本科高校等，加强职业技能培训师资队伍建设，支持紧缺领域人才培训。
　　13.广泛开展国际交流与合作。积极引入国（境）外相关领域职业标准、课程标准和技术标准，组织30所左右院校和企业引进国际先进课程设计和教学管理体系，结合我国国情和实际，开发本土化培养培训标准、方案、专业课程和教材。定期组织选派职业院校专业骨干教师赴国外研修访学。积极开展有关国际交流研讨活动。

　　**三、**实施保障
　　（一）加强部门协同
　　国务院教育行政部门在社会服务领域人才培养培训工作发挥牵头作用。有关行业主管部门、群团组织推动开展相关领域人才需求预测，指导专业设置和人才培养，引导行业优质企业积极参与产教融合、校企合作。省级教育行政部门要结合实际，高度重视社会服务产业紧缺人才培养培训工作，加强与省级有关部门的工作协同，健全工作机制。
　　（二）加大政策支持
　　各地教育行政部门要在政策、资金和项目等方面向积极开展社会服务产业紧缺领域人才培养培训的院校倾斜，会同有关部门落实好国家奖学金向家政、养老等社会急需专业倾斜的政策，吸引学生就读相关专业，保障相关专业家庭经济困难学生按照规定享受各类奖助政策，确保应助尽助。优先支持有关院校积极参与“家政服务业提质扩容‘领跑者’行动”等项目，促进社会服务产业高质量发展。支持建设若干集实践教学、社会培训、企业真实生产和社会技术服务于一体的高水平专业化产教融合实训基地。
　　（三）加强研究咨询
　　加强行业职业教育教学指导委员会等专家组织建设，提高行业指导能力，充分发挥专家组织的研究、咨询、指导、服务作用。设立一批社会服务产业紧缺领域教育研究项目，开展专题研究，为加快社会服务业人才培养提供理论支撑与智力支持。
　　（四）营造良好氛围
　　加强对社会服务产业新模式新业态、示范企业、特色院校、成长成才典型等的宣传，引导全社会和学生家长认识社会服务产业新定位、新理念、新职业，吸引相关专业毕业生对口就业，增强职业归属感、荣誉感。
　　（五）做好总结评价
　　各有关部门结合工作职责，将该意见相关任务落实情况于每年年底前及时总结，并提供教育部职业教育与成人教育司。省级教育行政部门要对本行政区域内有关专业设置、人才培养培训现状做全面摸底，研究设立有关工作项目，引导有关院校落实该意见各项任务，将社会服务产业领域相关专业设置、人才培养培训情况作为对有关院校绩效考核、质量评价的重要指标，支持第三方开展评估。

教育部办公厅
国家发展改革委办公厅
民政部办公厅
商务部办公厅
国家卫生健康委办公厅
国家中医药局办公室
全国妇联办公厅
2019年9月5日

© 北大法宝：（[www.pkulaw.com](https://www.pkulaw.com/)) 专业提供法律信息、法学知识和法律软件领域各类解决方案。北大法宝为您提供丰富的参考资料，正式引用法规条文时请与标准文本核对。
欢迎查看所有[产品和服务](https://www.pkulaw.com/helps/2.html)。法宝快讯：[如何快速找到您需要的检索结果？](https://www.pkulaw.com/helps/70.html)[法宝](https://www.pkulaw.com/helps/69.html)V6有何新特色？

扫描二维码阅读原文

**原文链接：**<https://www.pkulaw.com/chl/80a326cc4d6f2b8cbdfb.html>
